# Parasitic wasp females are attracted to blends of host-induced plant volatiles: do qualitative and quantitative differences in the blend matter?

**DOI:** 10.12688/f1000research.2-57.v2

**Published:** 2013-04-22

**Authors:** Masayoshi Uefune, Soichi Kugimiya, Rika Ozawa, Junji Takabayashi

**Affiliations:** 1 Center for Ecological Research, Kyoto University, Otsu, Shiga, 520-2113, Japan; 2 National Institute for Agro-Environmental Science (NIAES), Tsukuba, Ibaraki, 305-8604, Japan

## Abstract

Naïve *Cotesia vestalis* wasps, parasitoids of diamondback moth (DBM) larvae, are attracted to a synthetic blend (Blend A) of host-induced plant volatiles composed of sabinene, *n*-heptanal, α-pinene, and ( *Z*)-3-hexenyl acetate, in a ratio of 1.8:1.3:2.0:3.0. We studied whether qualitative (adding ( *R*)-limonene: Blend B) or quantitative changes (changing ratios: Blend C) to Blend A affected the olfactory response of *C. vestalis* in the background of intact komatsuna plant volatiles. Naïve wasps showed equal preference to Blends A and B and Blends A and C in two-choice tests. Wasps with oviposition experience in the presence of Blend B preferred Blend B over Blend A, while wasps that had oviposited without a volatile blend showed no preference between the two. Likewise, wasps that had starvation experience in the presence of Blend B preferred Blend A over Blend B, while wasps that had starved without a volatile blend showed no preference between the two. Wasps that had oviposition experience either with or without Blend A showed equal preferences between Blends C and A. However, wasps that had starvation experience in the presence of Blend A preferred Blend C over Blend A, while those that starved without a volatile blend showed equal preferences between the two. By manipulating quality and quantity of the synthetic attractants, we showed to what extent *C. vestalis* could discriminate/learn slight differences between blends that were all, in principle, attractive.

## Introduction

Plants infested by herbivorous insects release volatiles called herbivore-induced plant volatiles (HIPVs), which attract carnivorous natural enemies such as parasitic wasps and predators ^1– 3^. Blends of HIPVs differ from those of volatiles emitted by intact or artificially damaged plants and are specific to plant species, cultivars and developmental stage, as well as to herbivore species and developmental stage ^1– 3^. Natural enemies facilitate this specificity to find their victims. For example, parasitic wasps can distinguish between a blend of suitable host-induced plant volatiles and one of unsuitable host- or nonhost-induced plant volatiles to find a host ^4– 6^. Discrimination by the predatory mite between volatiles from plants infested with the prey and plants infested with the nonprey was also reported ^7^. These plant-specific responses by carnivores may be due to innate olfactory preferences or to olfactory learning of prey-infested plant volatiles ^4– 15^.

Learning is widespread among insects, and is relied on for all major activities ^16^. Parasitic wasps are well-established model systems for research on insect learning ^17^. Adult wasps can learn specific blends of HIPVs ^7– 13, 15^. For example, Fukushima *et al.* (2002) reported that *Cotesia kariyai* (Watanabe) (Hymenoptera: Braconidae), a parasitoid of *Mythimna separata* larvae (Lepidoptera: Noctuidae), that were preconditioned by simultaneous exposure to infested maize volatiles and host feces showed increased responses to a synthetic blend of five HIPVs of low specificity (i.e., induced by both artificial and host damage) ^8^. Takemoto *et al.* (2009) reported that *Aphidius ervi*, an aphid parasitoid, were not attracted to volatiles from host-infested broad bean plants over intact plant volatiles when they had emerged in clean Petri dishes, but when artificially exposed to infested-plant volatiles during emergence, the wasps showed a significant preference for infested-plant volatiles ^12^.

*Cotesia vestalis* is a specialist parasitoid of diamondback moth (DBM) ( *Plutella xylostella*) larvae, which feed on crucifer plants. We previously reported that naive *C. vestalis* were preferentially attracted to crucifer plants with DBM larval damage over artificially-damaged or nonhost ( *Pieris rapae* larvae)-infested plants ^6^. More recently, we reported that a blend of four compounds in the headspaces of DBM-infested cabbage plants attracted naive *C. vestalis* ^14^. The synthetic mixture was composed of sabinene, *n*-heptanal, *α*-pinene, and ( *Z*)-3-hexenyl acetate at a ratio of 1.8:1.3:2.0:3.0. The attractiveness of the synthetic blend was confirmed both under laboratory ^14, 18^ and field conditions ^18^.

An intriguing question about HIPV-mediated interactions between host-infested plants and natural enemies is to what extent natural enemies can distinguish qualitative and/or quantitative differences between two attractive volatile blends in combination with learning. To answer this question, we qualitatively or quantitatively manipulated the synthetic blend of volatiles that attracts *C. vestalis* to DBM-infested cabbage plants. By changing the ratio or adding another host-induced component to the artificial volatile blend, we tested whether these differences affected the olfactory responses of *C. vestalis* via learning of plant volatiles associated with hosts or foods. To our knowledge, this is the first report to show the extent to which wasps can recognize/learn qualitative or quantitative differences in volatile blends by using a synthetic blend of HIPVs attractive to *C. vestalis*.

## Materials and methods

### Insects and plants

Unless specified otherwise, all procedures described below were conducted in a climate-controlled room (25±3°C, 60±10% relative humidity [RH], 16-hour light: 8-hour dark [16L:8D] lighting schedule). Komatsuna ( *Brassica rapa* var. *perviridis* L. cv. Rakuten) plants (Takii & Co., Ltd., Kyoto Japan) were cultivated in soil (Ikubyou-baido: Takii & Co., Ltd., Kyoto Japan) in individual plastic pots (diameter: 90 mm, depth: 70 mm) for 4–5 weeks.

DBM were collected in a field near Kyoto, Japan, and mass-reared on potted komatsuna plants in the climate-controlled room. Eggs were collected every a few days, and hatched larvae were reared on cut plants in small cages (25 × 15 × 10 cm high).

*Cotesia vestalis* were obtained from parasitized DBM larvae collected in the same field. Newly-emerged adults were maintained in acryl cages (35 × 25 × 30 cm high) with 50% aqueous honey as food for 3 d to ensure mating. Female wasps were then individually transferred to glass tubes (2 cm diameter, 13 cm long) containing 50% aqueous honey as food and kept in a dark climate-controlled chamber (18±3°C, 60±10% RH, 24D) to suppress flight and prolong lifespan before use. Females were never kept for more than 6 d. At least 1 h before the start of each experiment, naive female wasps were transferred to the experimental chamber (25±3°C, 60±10% RH).

### Synthetic blend of HIPVs

The synthetic mixture of HIPVs was prepared as follows. Pure compounds of (Z)-3-hexenyl acetate (Wako Pure Chemical Industries, Ltd., Osaka, Japan), n-heptanal (Wako Pure Chemical Industries, Ltd., Osaka, Japan), α-pinene (Tokyo Chemical Industry Co. Ltd., Tokyo Japan, sabinene (Soda Aromatic Co. Ltd. Tokyo, Japan) and (R)-limonene (Wako Pure Chemical Industries, Ltd., Osaka, Japan) were dissolved in triethyl citrate (TEC) (Wako Pure Chemical Industries, Ltd., Osaka, Japan) (0.01% (w/w) TEC solution) to achieve slow volatilization. Using gas chromatography–mass spectroscopy (GCMS; Agilent 6890N/5973MSD System, Agilent, Santa Clara, CA, USA) with a TC-Wax capillary column (GL Sciences, Tokyo, Japan), the ratios of compounds in synthetic mixtures were adjusted to match those released by an infested cabbage plant ^14^. The gas chromatograph oven temperature was programmed to rise from 65°C to 120°C at 3°C/min, and then from 120°C to 245°C at 5°C/min. The synthetic mixture (Blend A) was composed of sabinene, n-heptanal, α-pinene, and (Z)-3-hexenyl acetate at a ratio of 1.8:1.3:2.0:3.0 ^14^. This blend was called Blend A.

We already reported that Blend A did not become more attractive to *C. vestalis* by adding ( *R*)-limonene, which was found in higher amounts in the headspaces of DBM-larvae-infested cabbage plants than intact ones ^14^. Thus, to test whether *C. vestalis* discriminated qualitative differences in volatile blends, we added limonene to make Blend B. The ratios of sabinene, *n*-heptanal, *α*-pinene, ( *Z*)-3-hexenyl acetate, and ( *R*)-limonene were adjusted to be 1.8:1.3:2.0:3.0:1.0 by GCMS. Next, to test whether female wasps could discriminate the ratios of the four compounds in the blend (quantitative differences in the blend), we prepared a third blend (Blend C) of sabinene, *n*-heptanal, *α*-pinene, and ( *Z*)-3-hexenyl acetate at a ratio of 1.0:1.0:1.0:1.0. In two-choice tests, we compared either Blends A vs. B or Blends A vs. C.

### Wasps’ conditioning

Female wasps that had experienced oviposition or starvation were prepared as follows. To generate oviposition-experienced *C. vestalis*, we confined a second stadium DBM larva in a glass vial (2 cm in diameter, 5 cm long), and then introduced a female *C. vestalis*. In most cases, oviposition occurred within 5 min. Wasps that oviposited on the larvae were used for bioassays. Starvation-experienced *C. vestalis* were then confined in a glass vial (2 cm in diam., 5 cm long) for 2 h without food.

To prepare wasps that had been exposed to volatiles during oviposition or starvation experience, we placed a piece of filter paper (1.5 × 1.5 cm) impregnated with 10 μL of a TEC solution of a volatile blend (see below) into the tube before confining a host larva and a wasp. For control (unexposed) experiments, we used a blank piece of filter paper of the same size. To compare the effects of added limonene to those of the original blend (Blend A), we used Blend B for the oviposition and starvation experiments. In the comparison of quantitatively-different blends (Blends A and C), we used Blend A for the oviposition and starvation experiments.

### Two-choice tests

Female *C. vestalis* were tested for their flight responses toward two potted intact komatsuna plants with a different blend. Two potted plants were placed in an acrylic cage (25 × 30 × 35 cm with three nylon-gauze–covered windows and one door) in a climate room at 25±2°C, 50–70% RH, and with continuous fluorescent light (20W, 3000 lux) without directed airflow ^6^. We placed a piece of filter paper (2 × 2 cm) impregnated with a 0.2 g blend in a Petri dish (diameter: 3.5 cm) at the base of the potted plants. Wasps were released individually from a glass tube positioned halfway between the plants. Each repeatedly hovered over the plants inside the cage, and when it first visited a plant (landed and initiated ambulatory search), it was removed with an aspirator. The plant visited was scored as its choice. For each replicate, usually 10 wasps were tested sequentially using the same pair of potted seedlings. Each treatment had three or four independent replicates. Female *C. vestalis* were used only once. New plants and blends were used for each replicate.

### Statistics

Two-choice data were analyzed using a replicated *G*-test ^19^. Wasps that chose neither plant were discarded from this analysis.

## Results

### Response of *cotesia vestalis* females to Blend A vs. Blend B

**Figure 1.**
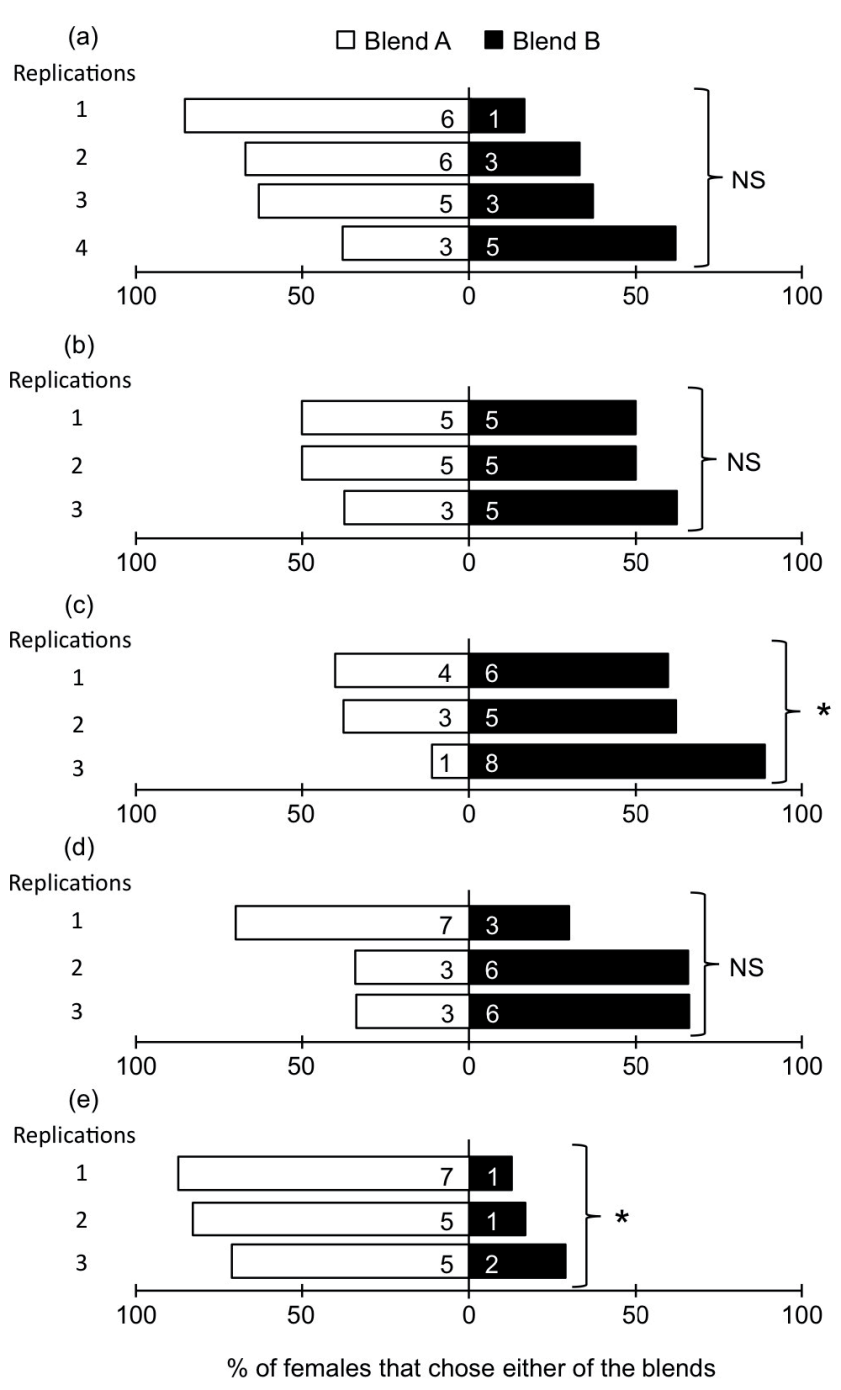
Responses of *Cotesia vestalis* to Blend A vs. Blend B. Each bar showed % of females that chose either of the blends (x axis). Numbers within bars indicate the numbers of females that landed on each plant. ( **a**) Response of naïve *C. vestalis*. ( **b**) Response of *C. vestalis* with oviposition experience. ( **c**) Response of *C. vestalis* with oviposition experience in the presence of Blend B. ( **d**) Response of *C. vestalis* with starvation experience. ( **e**) Response of *C. vestalis* with starvation experience in the presence of Blend B. *: 0.05 > *P* > 0.01, ns: not significantly different by G-test.

We first confirmed that oviposition-inexperienced (naïve) female wasps showed no significant preference between Blends A and B in the choice chamber (G-test, *Gt* = 5.9925, *P* = 0.1997; heterogeneity among samples: *Gh* = 3.9711, *P* = 0.2646; pooled effect of treatment: *Gp* = 2.0213, *P* = 0.1551) ( Figure 1a). When the female wasps had prior oviposition experience on hosts in the absence of synthetic blends, they showed an equal distribution between Blends A and B ( *Gt* = 1.2421, *P* = 0.7429; *Gh* = 1.2050, *P* = 0.5474; *Gp* = 0.0370, *P* = 0.8474) ( Figure 1b). However, when the female wasps had prior oviposition experience in the presence of Blend B, they showed a significant preference for Blend B over Blend A ( *Gt* = 7.9547, *P* = 0.0470; *Gh* = 2.6415, *P* = 0.2669; *Gp* = 5.3131, *P* = 0.0212) ( Figure 1c).

When the female wasps had experienced starvation in the absence of synthetic blends, they distributed equally between Blends A and B in the choice chamber ( *Gt* = 3.0677, *P* = 0.3813; *Gh* = 2.2013, *P* = 0.3326; *Gp* = 0.8664, *P* = 0.3520) ( Figure 1d). However, when they had been starved in the presence of Blend B, they significantly preferred Blend A over Blend B ( *Gt* = 9.3013, *P* = 0.0255; *Gh* = 0.6395, *P* = 0.7263; *Gp* = 8.6618, *P* = 0.0032) ( Figure 1e). Thus, both oviposition and starvation experience affected wasp preferences for Blend A or Blend B.

### Response of *cotesia vestalis* females to Blend A vs. Blend C

**Figure 2.**
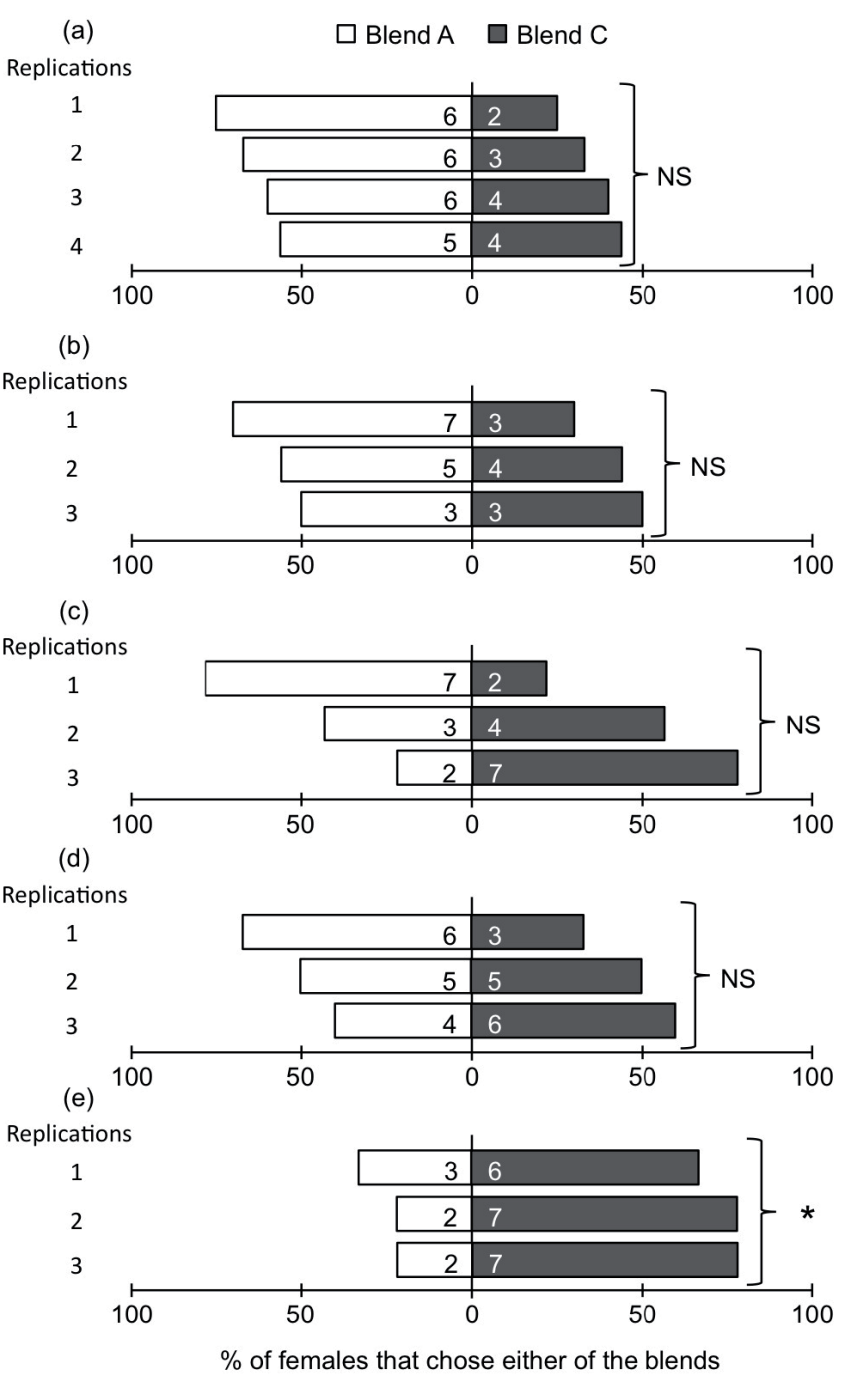
Responses of *Cotesia vestalis* to Blend A vs. Blend C. Each bar showed % of females that chose either of the blends (x axis). Numbers within bars indicate the numbers of females that landed on each plant. ( **a**) Response of naïve *C. vestalis*. ( **b**) Response of *C. vestalis* with oviposition experience. ( **c**) Response of *C. vestalis* with oviposition experience in the presence of Blend B. ( **d**) Response of *C. vestalis* with starvation experience. ( **e**) Response of *C. vestalis* with starvation experience in the presence of Blend B. *: 0.05 > *P* > 0.01, ns: not significantly different by G-test.

We first confirmed that naïve female wasps showed no significant preference between Blends A and C in the choice chamber ( *Gt* = 3.6264, *P* = 0.4589; *Gh* = 0.8118, *P* = 0.8466; *Gp* = 2.8147, *P* = 0.0934) ( Figure 2a). When the female wasps had previously oviposited in the absence of any synthetic blend, they distributed equally between Blends A and C in the choice chamber ( *Gt* = 1.7570, *P* = 0.6243; *Gh* = 0.7502, *P* = 0.6872; *Gp* = 1.0068, *P* = 0.3157) ( Figure 2b). Even when female wasps had oviposited in the presence of Blend A, they still showed no preference between Blends A and C in the choice chamber ( *Gt* = 6.0272, *P* = 0.1103; *Gh* = 5.987, *P* = 0.0501; *Gp* = 0.0400, *P* = 0.8415) ( Figure 2c).

When the female wasps had previously experienced starvation in the absence of any synthetic blend, they also distributed equally between Blends A and C in the choice chamber ( *Gt* = 1.4221, *P* = 0.7004; *Gh* = 1.3876, *P* = 0.4997; *Gp* = 0.0344, *P* = 0.8527) ( Figure 2d). However, when they experienced starvation in the presence of Blend A, they significantly preferred Blend C over Blend A ( *Gt* = 6.9032, *P* = 0.0750; *Gh* = 0.3765, *P* = 0.8284; *Gp* = 6.5268, *P* = 0.0106) ( Figure 2e). Thus, only starvation experience affected the choice between Blends A and C.

Raw data for figure 1 and figure 2 Raw data tables showing the number of female wasps that landed on each plant with the different blends (A,B or C).

## Discussion

We already reported the flight preference of *C. vestalis* to pure and mixed synthetic chemicals (Blend A) ^14^. When offered alone against pure solvent, none of the pure compounds elicited a significant preference in naive females of the parasitoid. However, when offered in mixtures against pure solvent, Blend A stands out as eliciting a significant preference. This mixture did not become more attractive by adding myrcene, camphor or limonene (compounds found to increase significantly in response to herbivory), and was just not significantly different in attractiveness from DBM-induced cabbage odor. Thus, Blend A triggered innate response in naive parasitoids, whereas the individual compounds did not. It was suggested that predatory mites, *Phytoseiulus persimilis*, did not recognize attractive HIPV in odor mixture but perceived odors as a synthetic whole ^11, 20^. Here, we showed to what extent parasitic wasps, *C. vestalis*, could recognize artificially created blend variation.

We manipulated the quality (Blend B) and quantity (Blend C) of the normal attractive synthetic blend (Blend A). The qualitative difference between Blends A and B was the presence in Blend B of ( *R*)-limonene, which is not attractive to *C. vestalis* ^14^, and the quantitative difference between Blends A and C was the ratio of the four compounds (1.8:1.3:2.0:3.0 vs. 1.0:1.0:1.0:1.0, respectively). Naïve wasps did not distinguish either qualitative or quantitative differences, suggesting that this wasp may have relatively broad responses to blends that contain these four essential compounds. By contrast, when the wasps had a positive experience (oviposition success) with a volatile blend, they could distinguish quality, i.e., they preferred the modified Blend B, but not quantitative differences, i.e., they preferred neither Blend A nor Blend C. Furthermore, when the wasps had a negative experience (starvation) in the presence of a volatile blend, they could distinguish both qualitative and quantitative differences. We believe this is the first study to show the extent to which parasitic wasps can identify qualitative and quantitative volatile differences by comparing naïve and tuned responses via associative learning.

In this study, the wasps experienced oviposition or starvation with or without volatile blends in glass tubes. Under natural conditions, parasitic wasps on host-infested plants would encounter not only HIPVs, but also host by-products such as feces. Fukushima *et al.* (2001) reported the ability of *C. kariyai* females to learn plant volatiles together with feces in a wind tunnel ^7^. The females experiencing host by-products together with the volatiles extracted from infested leaves for the first time showed an increased olfactory response. However, such behavioral changes were not observed in females that experienced only the host by-products or the volatiles. Whether multiple experiences of *C. vestalis* with host by-products together with HIPVs affects their olfactory responses to qualitatively and/or quantitatively different blends of HIPVs must be studied to clarify the mechanisms involved in plant-specific responses by parasitic wasps to HIPVs.
