# Supplementary material for: Parasitic wasp females are attracted to blends of host-induced plant volatiles: do qualitative and quantitative differences in the blend matter?
Source: F1000Res. 2013 Apr 22;2:57. Originally published 2013 Feb 21. [Version 2] doi: 10.12688/f1000research.2-57.v2 (PMC3829125; doi:10.12688/f1000research.2-57.v2)
Supplement: Peer review history

# Peer review: Referee response for version 2

**DOI:** 10.5256/f1000research.1385.r3980

**Published:** 2014-03-05

**Authors:** Yonggen Lou

**Affiliations:** 1 Institute of Insect Sciences, Zhejiang University, Hangzhou, China

I have read this submission. I believe that I have an appropriate level of expertise to confirm that it is of an acceptable scientific standard.

# Peer review: Referee response for version 2

**DOI:** 10.5256/f1000research.1385.r3212

**Published:** 2014-01-20

**Authors:** Jarmo Holopainen

**Affiliations:** 1 Chemical Ecology Research Group, University of Eastern Finland, Kuopio, Finland

I have read this submission. I believe that I have an appropriate level of expertise to confirm that it is of an acceptable scientific standard.

# Peer review: Referee response for version 1

**DOI:** 10.5256/f1000research.1227.r851

**Published:** 2013-03-18

**Authors:** Yonggen Lou

**Affiliations:** 1 Institute of Insect Sciences, Zhejiang University, Hangzhou, China

The paper written by Uefune *et al.* studied the effect of qualitative and quantitative changes in a synthetic blend on the behavioural response of *Cotesia vestalis*, a specialist parasitoid of diamondback moth. The experimental design and methods are correct, and have been clearly described. By bioassay, the authors found that the wasps could distinguish qualitative changes when they had a positive experience (oviposition success) with the volatile blend; while when they had a negative experience (starvation) with the blend, the wasps could distinguish both qualitative and quantitative differences. The result provides new insights into the role of associative learning in the host searching behavior of wasps, and also new information of the extent to which qualitative and quantitative changes can influence the behavioral responses of the parasitoid.  

**Minor concerns:**

-  It would be better to regard “ratio change” as a qualitative change; 
-  For both quantitative and qualitative changes, only one case was measured. It would be better to investigate more cases.   

I have read this submission. I believe that I have an appropriate level of expertise to confirm that it is of an acceptable scientific standard.

# Peer review: Referee response for version 1

**DOI:** 10.5256/f1000research.1227.r807

**Published:** 2013-03-04

**Authors:** Jarmo Holopainen

**Affiliations:** 1 Chemical Ecology Research Group, University of Eastern Finland, Kuopio, Finland

Uefune *et al. *have studied if qualitative or quantitative changes in the synthetic blend of the herbivore-induced plant volatiles will affect the olfactory response of *Cotesia vestali*s, a parasitoid of Diamondback moth. Their experiments are clearly described, carefully designed and conducted.  The results of this study show the significance of associative learning for the behaviour this wasp species. The results are also giving valuable new information of the extent to which parasitic wasps can identify qualitative and quantitative differences in herbivore-induced plant volatiles. When we know how to fine tune the associative learning in parasitic wasps that will improve the efficiency of the use of *C. vestalis* in biological control.

I have read this submission. I believe that I have an appropriate level of expertise to confirm that it is of an acceptable scientific standard.
